# Muscle hypertrophy and strength improvements following blood flow restriction combined with resistance training in team-athletes: a systematic review and meta-analysis

**DOI:** 10.3389/fphys.2026.1812707

**Published:** 2026-07-20

**Authors:** Qinshan Huang, Luohong Xiao, Songbo Zheng, Liuren Meng, Shun Yue

**Affiliations:** 1 College of Sports and Arts, JiangXi University of Science and Technology, Ganzhou, China; 2 Physical Education College, Jiangxi Normal University, Nanchang, China; 3 Nursing School, Youjiang Medical University for Nationalities, Baise, China; 4 Modern Service Academy, Hefei College of Finance and Economics, Hefei, China

**Keywords:** muscle adaptation, power, resistance training, team-athletes, vascular occlusion

## Abstract

**Objective:**

The purpose is to evaluate how blood flow restriction (BFR) and resistance training (RT), influence muscle adaptation and sports performance in team-athletes.

**Methods:**

Preferred Reporting Program for Systematic Reviews and Meta-analyses (PRISMA) guidelines are adhered to in the conduct of this study. From the beginning to September 11, 2025, a thorough literature search was conducted in PubMed, Web of Science, Cochrane Library, and SPORTDiscus. Subsequently, a random-effects meta-analysis was performed to synthesize data on muscle adaptation and sports performance.

**Results:**

The meta-analysis included 12 studies involving 859 team-athletes. Results indicated that, compared to RT alone, the addition of BFR significantly induced muscle hypertrophy (SMD = 0.32, 95% CI: 0.05 to 0.58, P = 0.02, I² = 4%) and improved muscle strength (SMD = 0.42, 95% CI: 0.18 to 0.66, P<0.001, I² = 10%). Jumping or sprint performance did not show any statistically significant differences.

**Conclusion:**

BFRT slightly improves the muscle hypertrophy and strength of team-athletes, but has no significant increase in explosive power output such as jumping and sprinting. It can be used as a complementary means of load management, but should be interpreted with caution. The optimal prescription for transforming it into explosive power should be explored in the future.

## 1 Introduction

BFR training begins with the rehabilitation and de-load phases of training (Sato, 2005; Slysz et al., 2016; Hughes et al., 2017). Controlled external pressure is applied to the limbs of participants, resulting in a partial restriction of arterial blood flow and a localized hypoxic environment within the muscle tissue (Hwang and Willoughby, 2019). This intervention leads to a rapid accumulation of metabolites, which in turn promotes fast-twitch muscle fiber recruitment (Suga et al., 2012; Cumming et al., 2014), exacerbates cell swelling (Loenneke et al., 2012; Freitas et al., 2017), and triggers a stronger hormonal and growth factor response (Fry et al., 2010; Patterson et al., 2013), supporting muscle adaptation. Its effects on participants are typically closely linked to the BFR technique, including exercise load, cuff pressure, cuff material and width, compression location, and duration (Ferguson et al., 2021; Rosenblatt et al., 2021).

In recent years, the application of BFR has gradually expanded to healthy athletes, and its positive effects on muscle adaptation have been observed (Scott et al., 2016; Pignanelli et al., 2021). Some evidence has been obtained in the field of sports science regarding the effects of BFR. The benefits of BFR for muscle adaptation have been comprehensively verified in various athlete populations, including weightlifters (Dobson, 2021), endurance athletes (Zhang et al., 2025; Ferguson et al., 2021; Xiao and Fu, 2025), college athletes (Zhang et al., 2025), and healthy athletes (Flocco and Galeoto, 2021). However, few studies currently focus on the application of BFR in team-athletes.

There is evidence that the combination of BFRT induces muscle adaptation in athletes, and that similar muscle gains can be achieved even with low loads as with high loads (Centner et al., 2023). BFRT is believed to create a stronger anabolic stimulus through the superposition of metabolic stress and mechanical tension, thereby bringing additional muscle gains to athletes (Wang et al., 2023). Previous studies have focused on low-load (20% - 50% 1RM) RT, with less attention paid to other loads in RT (Lixandrão et al., 2018). Recent reports indicate that, without differentiating between load types, BFRT results in significant increases in muscle morphology and strength among athletes in mixed sports (Scott et al., 2016; Wortman et al., 2021; Deng et al., 2025). This provides new application scenarios for athletes and coaches to use BFRT.

However, the specific effects of muscle improvement in certain athlete populations, such as team-athletes, remain unclear. The benefits of BFRT in team-athletes, particularly its impact on key sports performance such as sprinting and jumping, have not yet been clearly and comprehensively concluded. This limits the application of training programs in sports practice. Therefore, this study aims to clarify, i) the effects of BFRT on muscle hypertrophy and muscle strength in team-athletes, and ii) explore the comprehensive effects of the BFRT program on the sports performance of team-athletes, including jumping and short-distance sprint performance, so as to provide a training strategy basis for improving the muscle function and sports performance of team-athletes.

## 2 Methods

This meta-analysis was based on the 2020 PRISMA guidelines (Page et al., 2021). Prospective registration of the research protocol in the PROSPERO database (CRD420251144968) has been done.

### 2.1 Literature search

Searches were conducted on databases such as PubMed, Web of Science, Cochrane Library, and SPORTDiscus as of September 11, 2025. Keywords include “Blood flow restriction” “Kaatsu” “Ischemic training” “BFRT therapy” “BFRT” “Blood flow restriction training” “Blood flow restriction exercise” “Resistance training” “Strength training” “Weighted exercise program” “Team athlete” “Team member” “Team play” “Male team athlete” and “Female team athlete”. A manual search was performed by sifting through the references of the articles that were included. To guarantee that the literature search for this study was accurate, the two researchers conducted independent verification of the search terms. A third researcher (MLR) made the final decision after XLH and HQS disagreed on the search terms. A systematic review framework in sports science was employed to develop this strategy, which included discipline-specific terminology and optimized Boolean logic hierarchies to achieve accuracy, as illustrated in **Supplementary Table 1**.

### 2.2 Inclusion and exclusion criteria

The PICOS (Population, Intervention, Comparator, Outcomes, and Study Design) criteria listed in **Table 1** served as the basis for the inclusion and exclusion criteria in this meta-analysis. To ensure the caliber of the literature included in this analysis, only English literature with a PEDro score ≥ 6 (Albanese et al., 2020) was included.

**Table 1:** Inclusion and exclusion criteria for the literature.

Principle	Inclusion criteria	Exclusion criteria
P	Team-athletes, regardless of gender status	Team-athletes have clinical conditions such as sarcopenia, osteoarthritis, and cardiovascular disease
I	Regardless of the exercise load (e.g., low load, high load), RT programs should be used in conjunction with BFR	Other training methods or BFR combined with other training methods
C	Two-group or multi-group trials	Single-group trials
O	Team-athletes must have at least one outcome indicator, including lower limb muscle hypertrophy, Muscle strength, Jump performance, Sprint performance	Results that have nothing to do with these metrics or research that is unable to provide the mean and SD before and after the treatment
S	Randomized controlled trials (RCTs)	Non-RCTs
		Conference papers, systematic reviews (with or without meta-analysis), book chapters and reviews, and papers for which full text is unavailable
Other	—	

### 2.3 Coding of studies

All articles were subjected to a thorough evaluation following a review of the abstracts and titles. The following detail had been extracted: (i) participant characteristics, including age, sex, and sample size; (ii) a detailed description of the intervention, including training methods; (iii) specifics of the training variables; and (iv) the study outcomes. Conflicts were settled by argument until A contract had been reached, and a third worker (MLR) was consulted when there was disagreement. Outcomes such as lower limb muscle hypertrophy and strength, sprint performance, and jumping performance were collected during data collection.

### 2.4 Risk of bias assessments

To comprehensively assess the included articles’ methodical quality, researchers employed two instruments, including the PEDro (Physical Therapy Evidence Database) scale and the Cochrane Risk of Bias Tool Rob1.0. To measure methodological quality of the PEDro used, with items rated as either present (“1”) or absent (“0”), thus providing a comprehensive evaluation of each study. Studies that were included in this meta-analysis had to have received at least 6 points on the PEDro scale (Albanese et al., 2020) in order to guarantee the validity and reliability of the findings (**Table 2**). Additionally, the assessment matrix closely adheres to Chapter 8 of the Cochrane Handbook of Intervention Systems (Version 6.5), which covers 6 areas, including selection bias, performance bias, detection bias, attrition bias, reporting bias, and other biases. This is done using the Cochrane Risk of Bias Tool version 1.0 (RoB 1.0) in RevMan 5.1.4. Every study received one of three ratings: unknown risk (inadequate data to make a judgment), high risk (violates at least one important criterion), or low risk (meets at least two fundamental criteria) (Chandler et al., 2019).

**Table 2:** Study characteristics.

Study (year)	Sport & sex	Sample size (EG/CG)	Age (EG/CG)	Exercise protocol (type, duration, freq, load)	BFR parameters (pressure, width, mode)	Control	Outcomes
Scott et al., 2017	Football, M	18 (10/8)	19.8 ± 1.5/ 19.8 ± 1.5	Squat (5 weeks, 3x/w), 4 sets (15–30) @20–30% 1RM	7/10 PP, 7.5cm, Con-BFR	LL	CMJ↔, 10m↑, 20m↔, 40m↔, Muscle thickness↑
Castilla-López and Romero-Franco, 2023	Soccer, M	18 (NR)	19.22 ± 1.69/ 19.22 ± 1.69	Squat (6 weeks, 2x/w), 4 sets (15) @ 20-35% 1RM	40-80% AOP, 7cm, Int-BFR	HL	Girth↑, MVC↔, CMJ ↔, 30m↑
Yamanaka et al., 2012	Football, NR	32 (NR)	19.2 ± 1.8/ 19.2 ± 1.8	Bench press & Squat (4 weeks, 3x/w), 4sets (20-30) **@** 20%1RM	NR, 5cm, Con-BFR	No-BFR	1RM (Squat)↑, Girth(R/L)↑
Kamiş et al., 2024	Football, NR	24 (12/12)	19.25 ± 0.86/ 19.42 ± 1.24	Squat (8 weeks, 3x/w), 4sets (30-15-15-15) @20%1RM	40%-80%LOP, 10.5cm, Con‐BFR	No-BFR	Peak power↑, CMJ↔, 30m↑
Adhitya et al., 2022	Basketball/rugby, NR	43 (23/20)	16.3 ± 1.4/ 17.1 ± 2.3	Squat (8 weeks, 2x/w), 4 sets(10)@ 30%1RM	70%AOP, NR, Int‐BFR	No-BFR	Quadriceps strength↑
Gjini et al., 2024	Basketball, NR	18 (9/9)	21.44 ± 4.90/ 19.00 ± 3.96	Plantar flexion (4 weeks, 3x/w), 3 sets (3) @40%1RM	200mmHg, NR, Int‐BFR	No-BFR	Perimeter (R/L)↔, VJ↑
Wang et al., 2022	Volleyball, M	12 (6/6)	20.50 ± 1.38/ 20.83 ± 1.47	Squat (8 weeks, 3x/w), 4 sets (30-15-15-15) @30%1RM	50%AOP, 7cm, Con‐BFR	HL-RT	SJ↔
		12 (6/6)	20.17 ± 0.75/ 20.83 ± 1.47	Squat (8 weeks, 3x/w), 4 sets (30-15-15-15) @ 70%1RM			1RM (Half-squat) ↑, SJ↑
Golubev et al., 2021	Volleyball, M	18 (9/9)	18.7 ± 0.5/ 18.7 ± 0.5	Knee Extension & Squat (NR, 2x/w), 3 sets @25-40%1RM	400 SCU, 5cm, Con‐BFR	No-BFR	MVC↔
Manimmanakorn et al., 2013a	Football, F	20 (10/10)	20.2 ± 3.3/ 20.2 ± 3.3	Knee extension & flexion (5 weeks, 3x/w), 6 sets @20%1RM	230mmHg, 5cm, Con‐BFR	No-BFR	CSA↑, MVC3↑, VJ↑, 10m↔
Manimmanakorn et al., 2013b	Basketball, F	20 (10/10)	20.2 ± 3.3/ 20.2 ± 3.3	Knee extension & flexion (5 weeks, 3x/w), 6 sets @20%1RM	230mmHg, 5cm, Con‐BFR	No-BFR	CSA↑, MVC3↑
Smith et al., 2025	Basketball, F/M	17 (9/8)	21.1 ± 1.5/ 22.0 ± 2.1	Hard draw & Squat (4 weeks, 3x/w), 3 sets (10) @25-30%1RM	20-60%AOP, 10cm, Int‐BFR	No-BFR	Squat↑, CMJ↑, 5m↑, 10m↑, 20m↔
Korkmaz et al., 2022	Football/F	23 (11/12)	18.36 ± 0.5/ 18.42 ± 0.79	Quadriceps extension (6 weeks, 2x/w), 4 sets (30-15-15-15) @30%1RM	130–150mmHg, 7cm, Con‐BFR	No‐BFRHL	Muscle thickness↑, Peak knee torque↑

EG, Experimental Group; CG, Control Group; M, Male; F, Female; NR, Not Reported; BFR, Blood Flow Restriction; Con-BFR, Continuous BFR; Int-BFR, Intermittent BFR; AOP, Arterial Occlusion Pressure; LOP, Leg Occlusion Pressure; PP, Perceived Pressure; LL, Low Load; HL, High Load; CMJ, Countermovement Jump; VJ, Vertical jump; SJ, Squat Jump; MVC, Maximal Voluntary Contraction; 1RM, One-repetition maximum; CSA, Cross-sectional area; ↑ indicates significant improvement; ↔ indicates no significant difference compared to baseline or control.

### 2.5 Data synthesis and statistical analysis

This study used a random-effects model to calculate the pooled effect size, regardless of observed heterogeneity. Its purpose was to account for potential clinical and methodological differences among studies. To quantify the magnitude of the intervention effect, we calculated the standardized mean difference (SMD) for all continuous outcome measures using the following method (Orange et al., 2022):


$$\mathrm{SMD}=\frac{{\text{Mean}}_{\text{change-intervention}}-{\text{Mean}}_{\text{change-control}}}{\text{pooled\_SD\_change}}$$

The calculation method for Mean _change_ is as follows (Orange et al., 2022; Zhou et al., 2024):


$${\text{Mean}}_{\text{change}}={\text{Mean}}_{\text{final}}-{\text{Mean}}_{\text{baseline}}$$

Mean _change_ is the mean change, Mean _final_ is the post-test mean, Mean _baseline_ is the baseline mean, Mean _change-intervention_ is the mean change before and after treatment in the experimental group, and Mean _change-control_ is the mean change before and after treatment in the control group. Pooled SD _change_ is the pooled standard deviation of the change scores. If the SD of the change score was not reported and could not be retrieved from the corresponding author, it was estimated from the baseline and post-intervention SD _change_ using the following formula (Orange et al., 2022; Zhou et al., 2024):


$${\text{SD}}_{\text{change}}=\sqrt{{{\text{SD}}^{2}}_{\text{baseline}}+{{\text{SD}}^{2}}_{\text{final}}-2\text{r}\times {\text{SD}}_{\text{baseline}}\times {\text{SD}}_{\text{final}}}$$

If the r was not reported and could not be retrieved from the corresponding author, it was estimated from the baseline and post-intervention SD _change_ using the following formula:


$$r=\frac{S{D^{2}}_{\text{baseline}}+S{D^{2}}_{\text{final}}-S{D^{2}}_{\text{change}}}{2\times SD_{\text{baseline}}\times SD_{\text{final}}}$$

where r represents the pre-post correlation coefficient. This approach is consistent with Cochrane guidance, which states that when the SD of the change from baseline is missing but baseline and post-intervention SD _change_ are available, the missing change-score SD can be imputed using a calculated, externally imputed, or hypothesised correlation coefficient (Higgins and Deeks, 2024). In the absence of study-specific pre-post correlations, we assumed a correlation coefficient of 0.7, because this value has been used as a conservative assumption in previous meta-analyses when pre-post correlations were not reported (Orange et al., 2021). Estimates based on imputed change-score SD _change_ were therefore interpreted with caution.

To minimize potential bias from small sample sizes, we applied Hedges’ g-correction to the SMD. Effect sizes were interpreted according to Cohen (1988) criteria, including negligible (<0.2), small (0.2–0.49), moderate (0.5–0.79), and large (≥0.8)( Cohen, 1988) We assessed statistical heterogeneity among included studies using the Q statistic and I² statistic. I² values of 25%, 50%, and 75% represented low, moderate, and high heterogeneity, respectively (Higgins et al., 2003).

To assess the robustness of the pooled estimates, a sensitivity analysis was performed using a leave-one-out method. This method sequentially eliminated each study from the meta-analysis to determine if any single study had an excessively large impact on the overall effect size or level of heterogeneity. Furthermore, to verify the stability of the results, a fixed-effects model analysis was performed as an auxiliary analysis to compare its consistency with the results of the random-effects model.

Potential publication bias was assessed using statistical methods. Funnel plots were plotted to visually examine the symmetry of the study effect size distribution. Furthermore, Egger’s linear regression test was performed to quantitatively assess plot asymmetry. A p-value<0.05 in the Egger’s test was considered to indicate significant publication bias.

The meta-analysis of this study used Stat MP 17.0 (Version 17.0, Stata Corp LLC, College Station, USA) and Review Manager (Version 5.4.1, Cochrane Collaboration, Copenhagen, Denmark) software. Literature screening was performed using Note Express (Version 4.2.0, Beijing Virginidove Technology Co., Ltd.). p< 0.05 was set as statistical significance. Data are presented as point estimates and their corresponding 95% CI. Positive effects favored the experimental group, and negative effects favored the control group. One author (HQS) performed the statistical analysis, and another author (XLH) checked the code and reproduced the results.

## 3 Results

### 3.1 Search results

**Figure 1:**
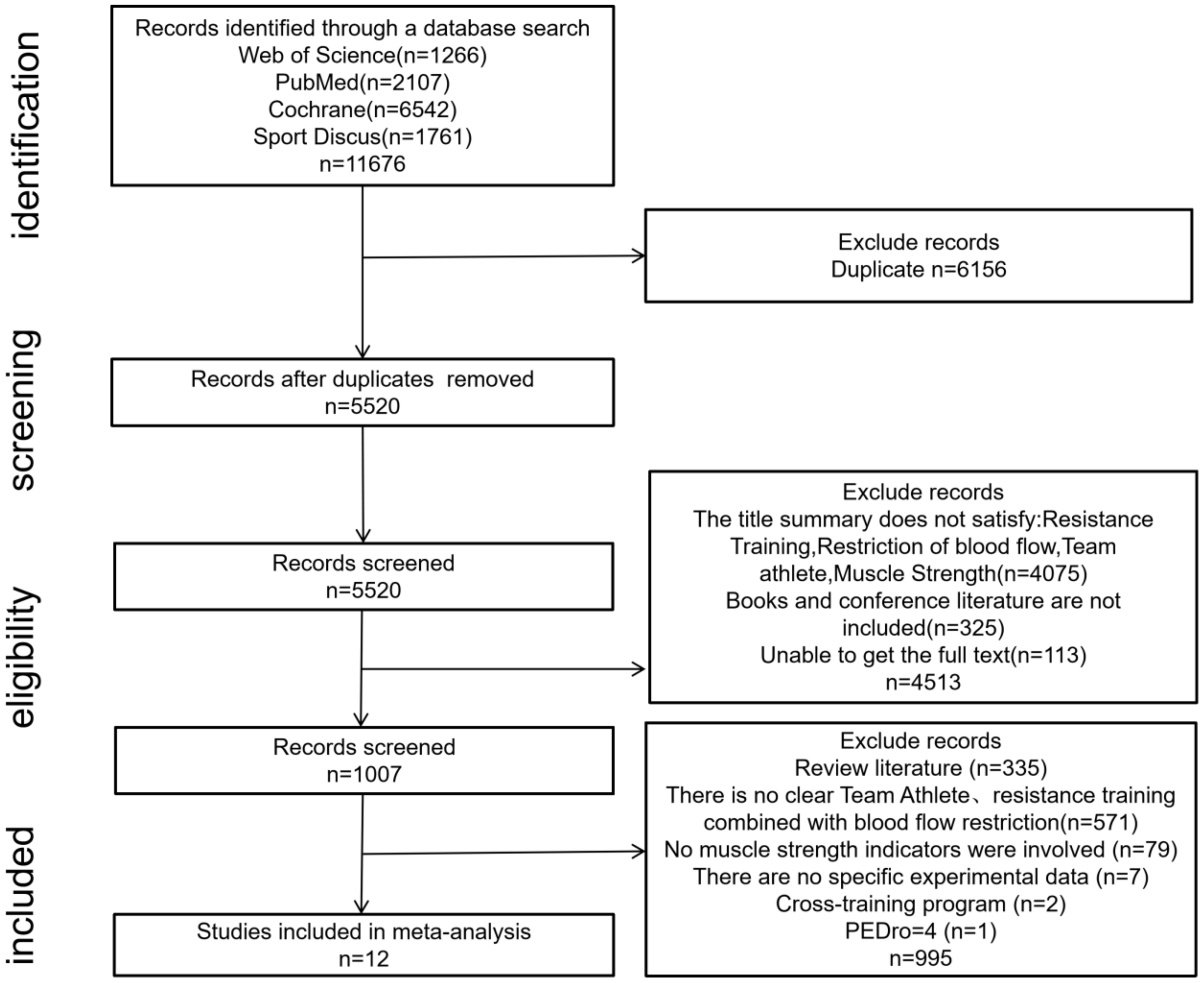
Flowchart illustrating a systematic review process with stages of identification, screening, eligibility, and inclusion; records are narrowed from 11,676 to 12 studies included in meta-analysis through exclusions for duplicates, relevance, and methodological criteria.

11, 676 articles were retrieved through relevant literature searches, and were further filtered according to the research object and purpose of this paper. Using Note Express software, 6156 duplicate articles were removed, leaving 5520 articles. A preliminary screening was carried out using the title and summary after duplicate content was eliminated. After that, the complete text was examined and rescreened to exclude studies that didn’t fit the requirements for inclusion. In the end, 12 studies were included in the meta-analysis. **Figure 1** depicts the thorough search procedure used for this investigation.

### 3.2 Research quality risk assessment

**Figure 2:**
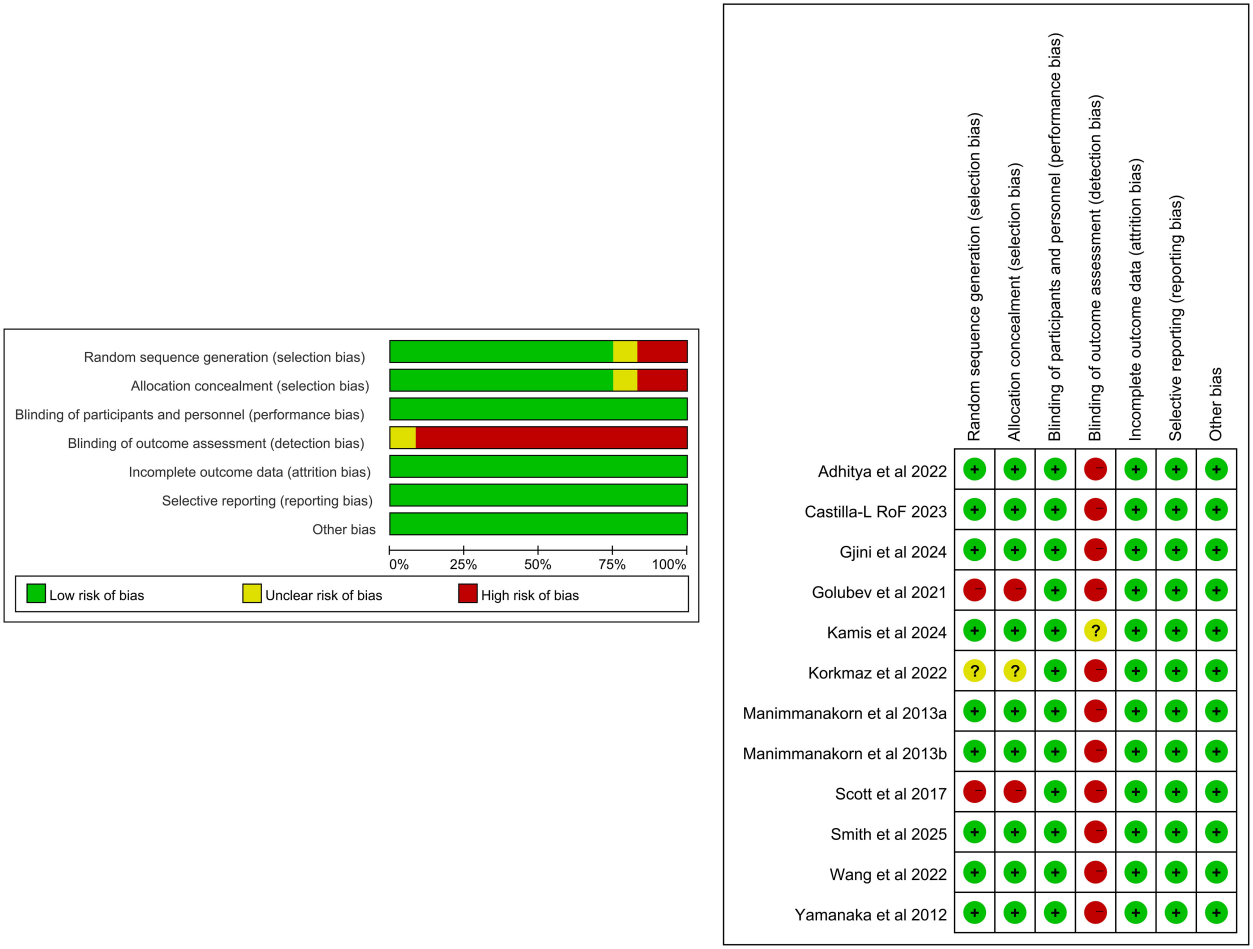
Bar chart and dot matrix summarizing risk of bias across seven study domains, using green for low risk, yellow for unclear risk, and red for high risk. Most domains have predominantly low risk, except “Blinding of outcome assessment” and “Blinding of participants and personnel”, which show higher proportions of high risk. The dot matrix maps individual studies to each bias domain using color-coded circles matching the bar chart, with a legend explaining the color scheme.

Two researchers (XLH and HQS) independently evaluated the quality of each study included in this meta-analysis. **Figure 2** displays the findings of the quality evaluation of the 12 included RCTs, which was conducted using the Cochrane risk of bias tool criteria. 11 studies (91.7%) had a high risk of measurement bias (evaluators knew the evaluation indicators), while 1 study (8.3%) had a moderate risk of measurement bias in its method, as reported by the risk assessment tool. The included studies were all at low risk of experimental bias, follow-up bias, reporting bias, and other biases (lack of blinding of researchers and participants and no impact on experimental results, missing participants not affecting the experiment, reporting of outcome indicators, and uniformity of testing tools), therefore the quality of the included studies was moderate.

Meanwhile, the PEDro scale scores included in this study were 7.25 ± 1.09 (**Table 3**), with 9 studies (75%) scoring ≥ 7 points and 3 studies (25%) scoring 6 points. Notably, the 3 studies with scores of 6 points were assessed as having a risk of bias. These specific studies may contribute to the observed heterogeneity and may affect the overall robustness of the results.

**Table 3:** PEDro quality assessment of the included studies.

References	2	3	4	5	6	7	8	9	10	11	Total (from a possible maximal of 10)
Scott et al., 2017	0	1	1	0	0	0	1	1	1	1	6
Castilla-López and Romero-Franco, 2023	1	1	1	0	0	1	1	1	1	1	8
Yamanaka et al., 2012	1	1	1	0	0	0	1	1	1	1	7
Kamiş et al., 2024	1	1	1	0	0	0	1	1	1	1	7
Adhitya et al., 2022	1	1	1	0	0	1	1	1	1	1	8
Gjini et al., 2024	1	1	1	1	1	1	1	1	1	1	10
Wang et al., 2022	1	1	1	0	0	0	1	1	1	1	7
Golubev et al., 2021	0	1	1	0	0	0	1	1	1	1	6
Manimmanakorn et al., 2013a	1	1	1	0	0	0	1	1	1	1	7
Manimmanakorn et al., 2013b	1	1	1	0	0	1	1	1	1	1	8
Smith et al., 2025	1	0	1	1	0	0	1	1	1	1	7
Korkmaz et al., 2022	0	0	1	0	0	1	1	1	1	1	6

Median Score = 7.25.

### 3.3 Study characteristics

Following screening studies, the whole text was used to evaluate all pertinent eligibility. At this point, we extracted pertinent population characteristics, BFR features, training protocols, and primary outcome measures. Only the final time point (as a post-training value) is taken into account when evaluating the intervention’s efficacy at various time points. We get in touch with the manuscript’s associated author if the original data is lacking. The data is deleted if the author cannot be contacted. Two researchers (XLH and HQS) independently assessed each study for inclusion in this systematic review using the data that was extracted. **Table 2** displays the data that was gathered from the included research.

This meta-analysis includes 12 articles (Adhitya et al., 2022; Castilla-López and Romero-Franco, 2023; Gjini et al., 2024; Golubev et al., 2021; Kamiş et al., 2024; Korkmaz et al., 2022; Manimmanakorn et al., 2013a, 2013; Scott et al., 2017; Smith et al., 2025; Wang et al., 2022; Yamanaka et al., 2012) and 859 team-athletes published between 2002 and 2025. Each study had a sample size of 6 to 23 participants aged 16.3 to 25 years. 4 studies focused specifically on male team-athletes, 3 on female team-athletes, 1 included both, and 4 did not report gender. The sample covered 5 sports: volleyball, football, netball, Rugby, and basketball (**Table 2**).

Current BFRT programs is based on continuous or intermittent flow restriction pressure, with different pressures producing varying effects. Training frequencies varied between 2 and 3 times per week across the 12 included studies. Resistance loads ranged from 20% - 70% 1RM. In all studies, the intensity of the exercise intervention was mentioned in all trial groups, and the number of repetitions per training session was specifically reported for each intervention group.

This study included trial groups that used different types of tourniquets. 10 studies (Castilla-López and Romero-Franco, 2023; Golubev et al., 2021; Kamiş et al., 2024; Korkmaz et al., 2022; Manimmanakorn et al., 2013a, 2013; Scott et al., 2017; Smith et al., 2025; Wang et al., 2022; Yamanaka et al., 2012) described specific details of the tourniquets, while 2 studies (Adhitya et al., 2022; Gjini et al., 2024) did not report specific details.

### 3.4 Analysis of team-athletes’ muscle function and sports performance

#### 3.4.1 Muscle hypertrophy

**Figure 3:**
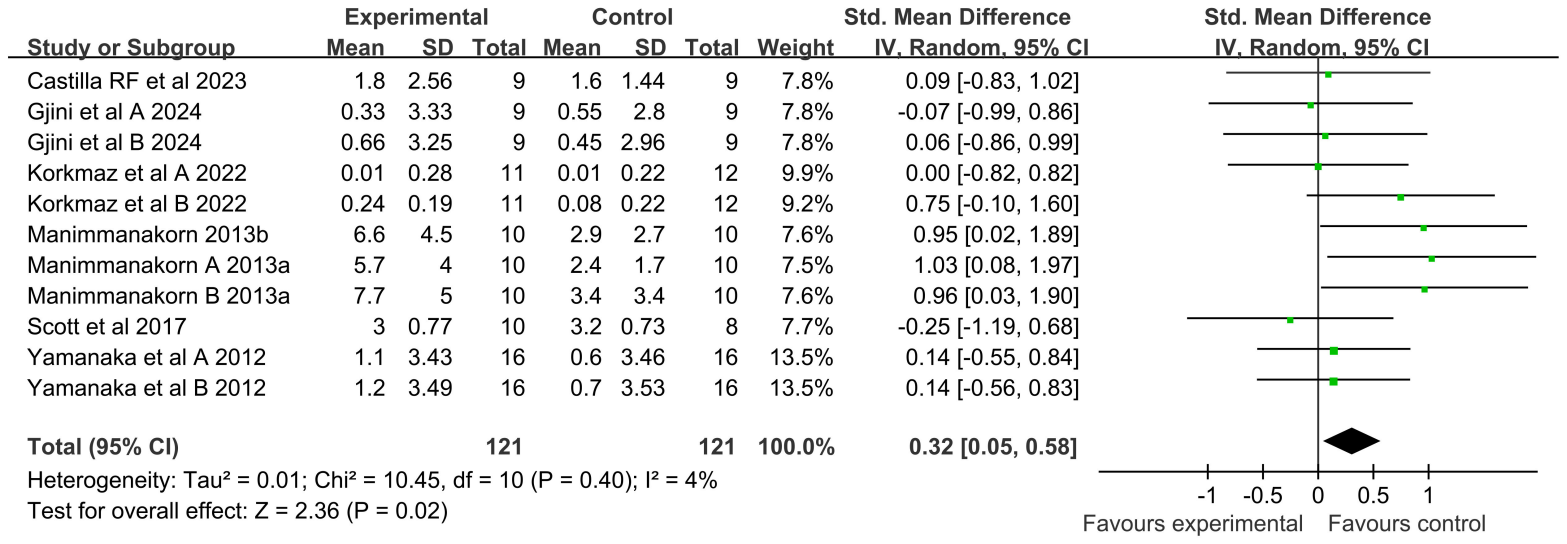
Forest plot summarizing a meta-analysis of eleven studies comparing experimental and control groups, showing standardized mean differences with confidence intervals. The overall effect favors the experimental group with an effect size of 0.32 [0.05, 0.58], low heterogeneity, and a test for overall effect Z = 2.36 *P* = 0.02.

As shown in **Figure 3**, a total of 7 original studies were included, encompassing 11 outcomes and 242 participants. The meta-analysis showed a SMD of 0.32, with a 95% CI ranging from 0.05 to 0.58. The interval width was close to 0.5, indicating a relatively precise pooled effect (p = 0.02). Despite clinical differences in treatment regimens among the original studies, no significant statistical heterogeneity was found (Cochran’s Q = 10.45, I² = 4%, p = 0.40).

#### 3.4.2 Muscle strength

**Figure 4:**
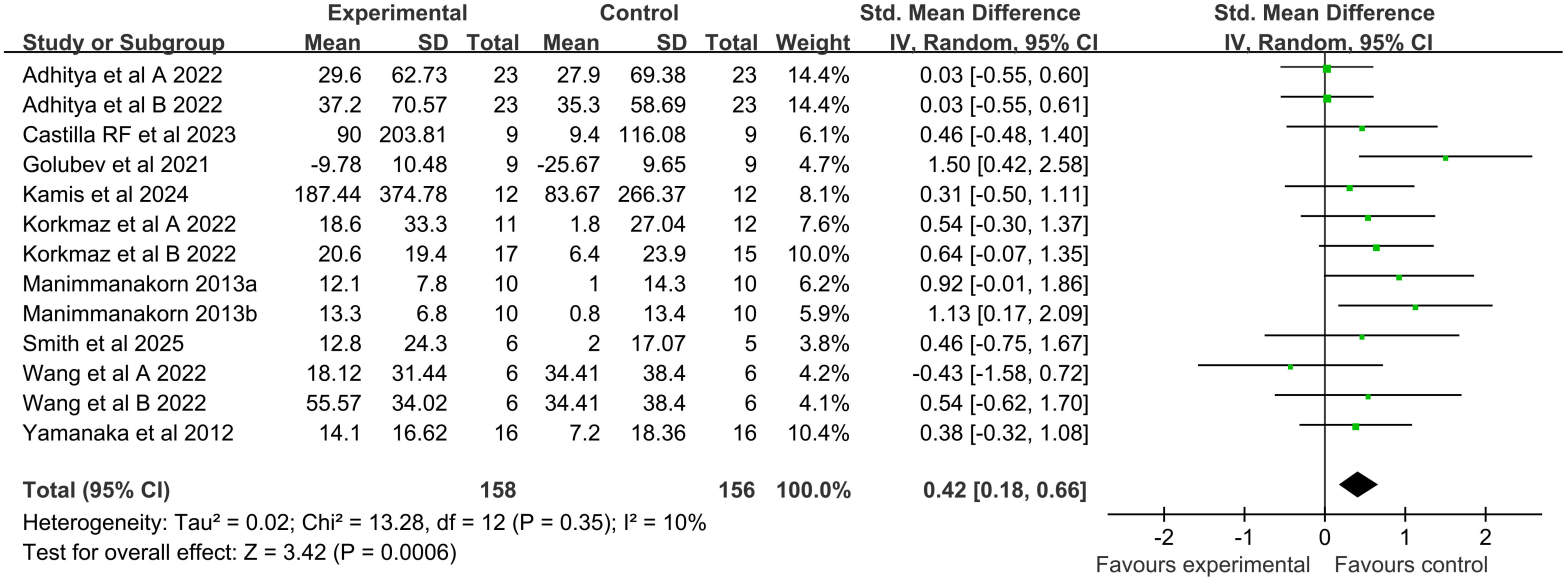
Forest plot displaying a meta-analysis comparing standardized mean differences between experimental and control groups across thirteen studies, with confidence intervals shown as horizontal lines. The summary effect size is 0.42, favoring the experimental group, with low heterogeneity reported.

As shown in **Figure 4**, a total of 9 original studies were included, encompassing 13 outcomes and 311 participants. The meta-analysis showed a SMD of 0.42, with a 95% CI ranging from 0.18 to 0.66, an interval width less than 0.5, and a precise pooled effect (p< 0.001). Despite clinical differences in treatment regimens among the original studies, no significant statistical heterogeneity was found (Cochran’s Q = 13.28, I² = 10%, p = 0.35).

#### 3.4.3 Jump performance

**Figure 5:**
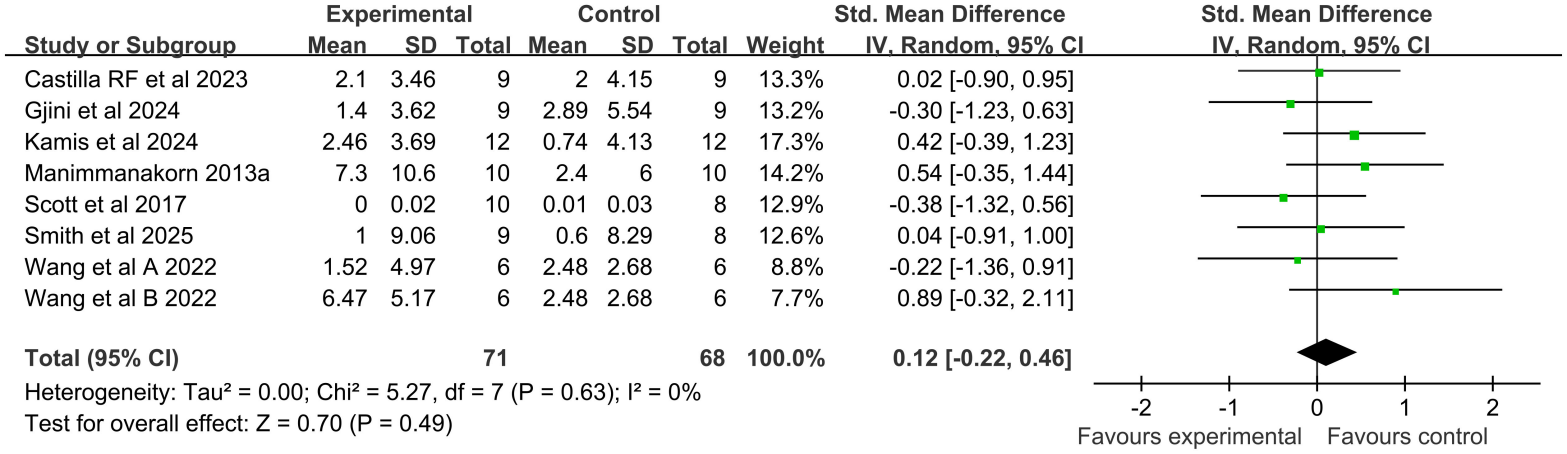
Forest plot summarizing eight studies comparing experimental and control groups for standard mean difference. Green squares indicate individual study results with confidence intervals; the overall effect size is 0.12 with confidence interval from negative 0.22 to 0.46, favoring neither group.

As shown in **Figure 5**, a total of 7 original studies were included, with 8 outcomes and 139 participants. The meta-analysis showed that the SMD was 0.12, the 95% CI ranged from -0.22 to 0.46, the interval width was greater than 0.5, and the pooled effect was imprecise (p=0.49).

#### 3.4.4 Sprint performance

**Figure 6:**
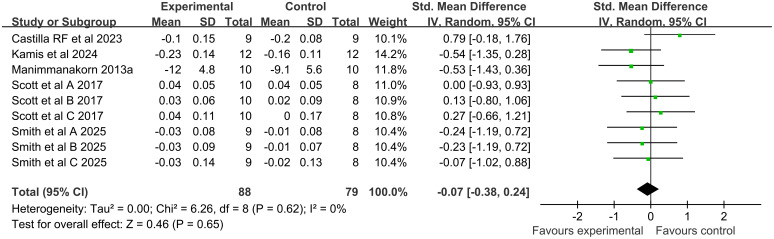
Forest plot of nine studies comparing experimental and control groups, showing standardized mean differences with confidence intervals. Pooled result is negative zero point zero seven, indicating no significant effect. All confidence intervals cross zero.

As shown in **Figure 6**, a total of 5 original studies were included, with 9 outcomes and 167 participants. The meta-analysis showed that the SMD was 0.07, the 95% CI ranged from -0.38 to 0.24, the interval width was greater than 0.5, and the pooling effect was imprecise (p=0.65).

### 3.5 Publication bias

**Figure 7:**
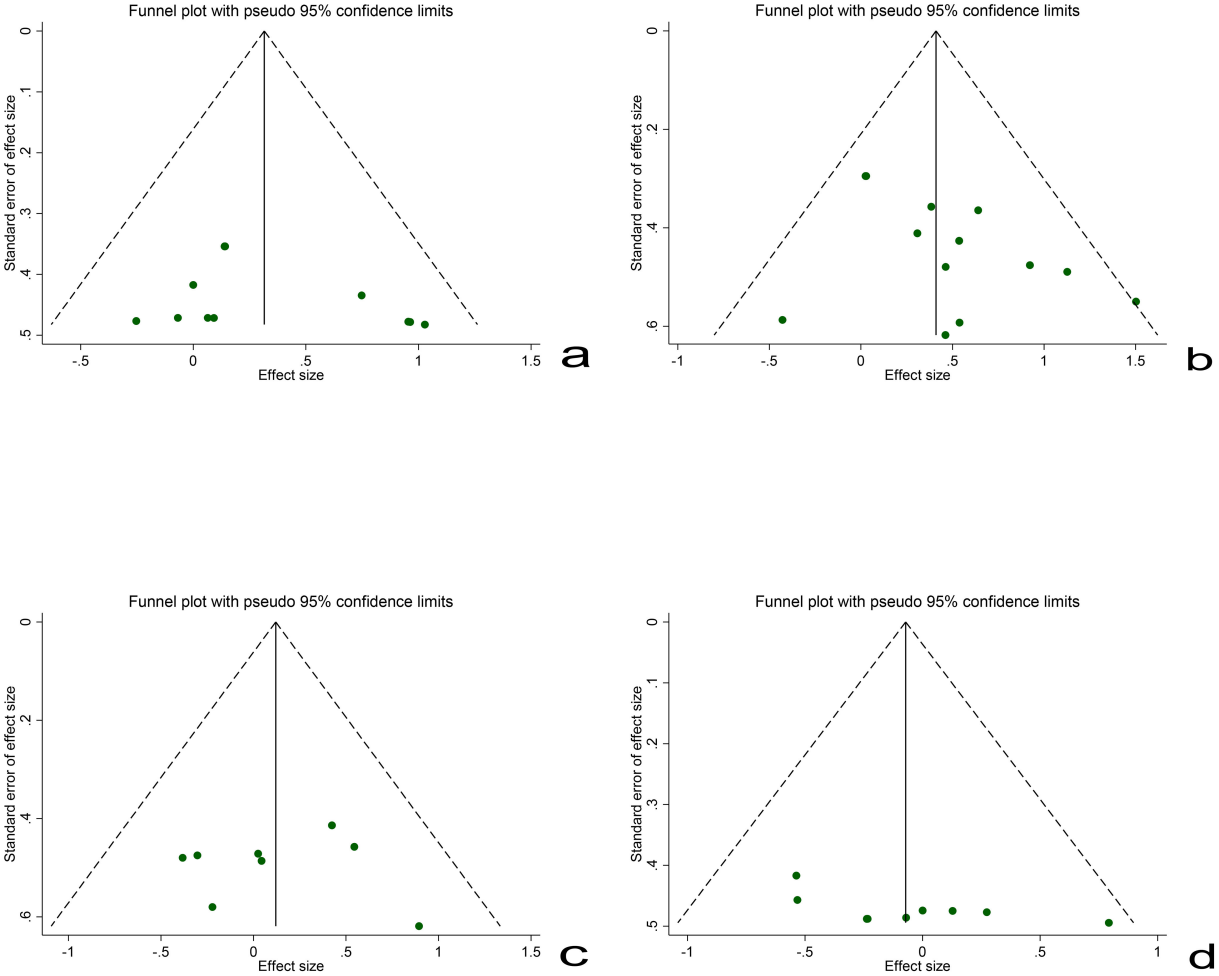
Four-panel figure showing funnel plots labeled a, b, c, and d, each with effect size on the x-axis and standard error of effect size on the y-axis. Each plot contains a triangle formed by dashed lines representing pseudo ninety-five percent confidence limits, along with green dots representing data points scattered within each triangle. Plots **(b, d)** contain more data points than **(a, c)**. Each plot visually assesses publication bias or heterogeneity in a meta-analysis context. **(a)**: funnel plots of Muscle hypertrophy, **(b)**: funnel plots of Muscle strength, **(c)**: funnel plots of Jump performance, (d): funnel plots of Sprint performance.

**Figure 8:**
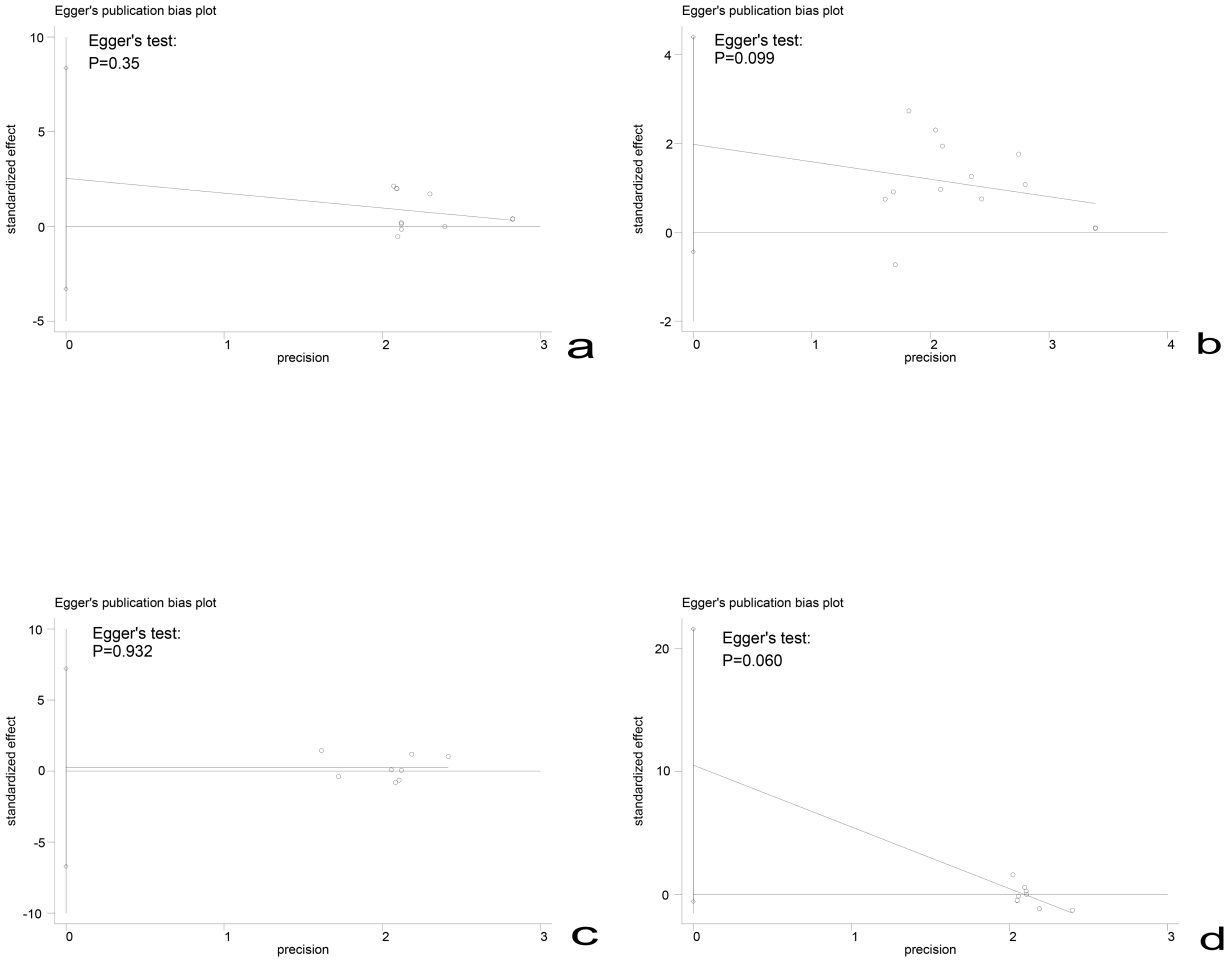
Four-panel graphic showing Egger’s publication bias plots with regression lines. Panel **(a)**: P equals 0.35; panel **(b)**: P equals 0.099; panel **(c)**: P equals 0.932; panel **(d)**: P equals 0.06. Each plot depicts standardized effect against precision with no significant publication bias indicated. **(a)**: Egger’s publication bias plots of Muscle hypertrophy, **(b)**: Egger’s publication bias plots of Muscle strength, **(c)**: Egger’s publication bias plots of Jump performance, **(d)**: Egger’s publication bias plots of Sprint performance.

The funnel plot results show that muscle hypertrophy, strength, jumping, and sprinting are all symmetrically distributed (**Figure 7**). Egger results showed no evidence of publication bias in muscle hypertrophy (t = 0.98, P = 0.35) (**Figure 8a**), muscle strength (t = 1.8, P = 0.099) (**Figure 8b**), jumping (t = 0.09, P = 0.932) (**Figure 8c**) and sprinting (t = 2.24, P = 0.06) (**Figure 8d**).

### 3.6 Sensitivity analysis

Leave-one-out sensitivity analysis assesses the robustness of pooled effect sizes and examines the impact of individual studies on the overall outcome (Zou et al., 2026).

**Figure 9:**
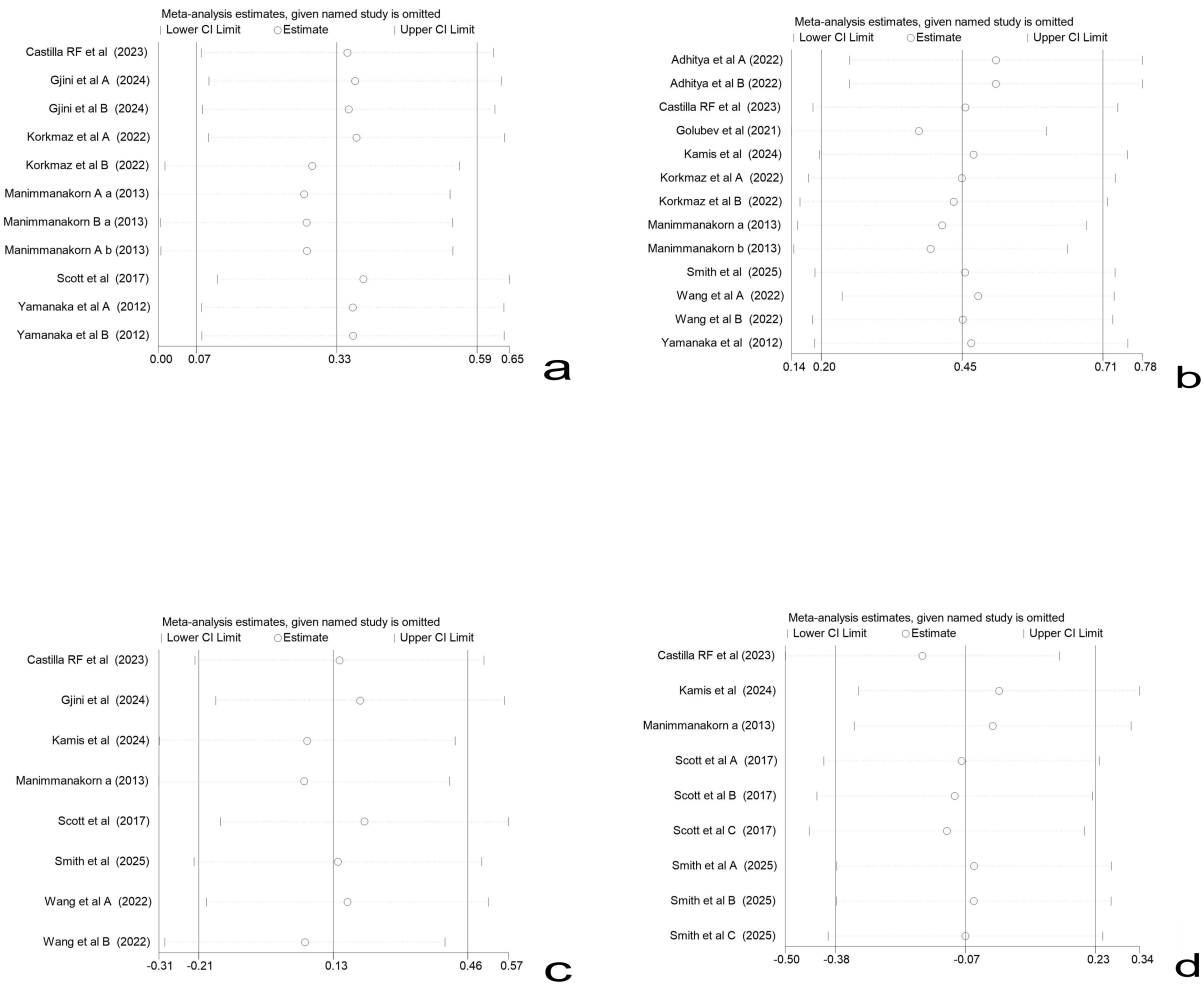
Four-panel figure showing leave-one-out meta-analysis plots labeled a, b, c, and d. Each panel lists individual studies on the y-axis and displays point estimates with lower and upper confidence intervals along the x-axis. Each plot visually assesses the influence of omitting one study at a time on the summary effect estimate, with circles representing estimates and horizontal lines for confidence intervals. The panels are arranged in a two-by-two grid and each study is identified by author and year. **(a)**: leave-one-out meta-analysis plots of Muscle hypertrophy, **(b)**: leave-one-out meta-analysis plots of Muscle strength, **(c)**: leave-one-out meta-analysis plots of Jump performance, **(d)**: leave-one-out meta-analysis plots of Sprint performance.

The results on muscle hypertrophy showed that no single study had a dominant effect on the pooled effect size (**Table 4**). The sum of magnitudes (SMD) ranged from 0.27 to 0.38, and all 95% confidence intervals overlapped with the original pooled effect size (SMD = 0.33, 95% CI: 0.07 to 0.59). The effect size remained positive in all exclusion scenarios. The most significant changes were observed after excluding Korkmaz et al. (2022) and Scott et al. (2017), with pooled effect sizes of 0.28 (95% CI: 0.01 to 0.55) and 0.38 (95% CI: 0.11 to 0.65), respectively (**Figure 9a**).

**Table 4:** Leave-one-out sensitivity analysis for muscle hypertrophy.

Study omitted	Estimate	95 % Conf. interval	
		Lower CI limit	Upper CI limit
Castilla-López and Romero-Franco (2023)	0.349	0.081	0.617
Gjini et al. (2024)	0.363	0.095	0.631
Gjini et al. (2024)	0.351	0.083	0.619
Korkmaz et al. (2022)	0.365	0.094	0.636
Korkmaz et al. (2022)	0.284	0.014	0.554
Manimmanakorn et al. (2013a)	0.269	0.002	0.537
Manimmanakorn et al. (2013b)	0.274	0.006	0.541
Manimmanakorn et al. (2013a)	0.274	0.006	0.542
Scott et al. (2017)	0.378	0.110	0.646
Yamanaka et al. (2012)	0.358	0.081	0.635
Yamanaka et al. (2012)	0.359	0.082	0.636
Combined	0.329	0.072	0.586

The muscle strength results showed that no single study had a dominant effect on the pooled effect size (**Table 5**). The sum of magnitudes (SMD) ranged from 0.37 to 0.51, and the confidence intervals all overlapped with the original pooled effect sizes (SMD = 0.45, 95% CI: 0.19 to 0.70). The pooled effect size decreased most significantly after removing Golubev et al. (2021) (SMD = 0.37, 95% CI: 0.13 to 0.60), and increased most significantly after removing Adhitya et al. (2022) (SMD = 0.51, 95% CI: 0.24 to 0.78) (**Figure 9b**).

**Table 5:** Leave-one-out sensitivity analysis of muscle strength.

Study omitted	Estimate	95 % Conf. interval	
		Lower CI limit	Upper CI limit
Adhitya et al. (2022)	0.514	0.247	0.781
Adhitya et al. (2022)	0.514	0.247	0.781
Castilla-López and Romero-Franco (2023)	0.458	0.181	0.736
Golubev et al. (2021)	0.374	0.141	0.606
Kamiş et al. (2024)	0.473	0.192	0.754
Korkmaz et al. (2022)	0.452	0.172	0.732
Korkmaz et al. (2022)	0.437	0.157	0.717
Manimmanakorn et al. (2013a)	0.416	0.153	0.679
Manimmanakorn et al. (2013b)	0.395	0.146	0.644
Smith et al. (2025)	0.458	0.184	0.731
Wang et al. (2022)	0.482	0.234	0.730
Wang et al. (2022)	0.453	0.180	0.727
Yamanaka et al. (2012)	0.469	0.184	0.754
Combined	0.453	0.196	0.709

The jump results showed that no single study had a dominant effect on the pooled effect size (**Table 6**). The original pooled effect size was not statistically significant (SMD = 0.13, 95% CI: -0.21 to 0.46). After eliminating each study individually, the SMD ranged from 0.05 to 0.20, with all confidence intervals including 0 (**Figure 9c**).

**Table 6:** Leave-one-out sensitivity analysis of jump performance.

Study omitted	Estimate	95 % Conf. interval	
		Lower CI limit	Upper CI limit
Castilla-López and Romero-Franco (2023)	0.142	-0.220	0.504
Gjini et al. (2024)	0.194	-0.168	0.556
Kamiş et al. (2024)	0.061	-0.310	0.432
Manimmanakorn et al. (2013a)	0.054	-0.311	0.418
Scott et al. (2017)	0.205	-0.157	0.566
Smith et al. (2025)	0.138	-0.223	0.499
Wang et al. (2022)	0.162	-0.191	0.515
Wang et al. (2022)	0.055	-0.296	0.407
Combined	0.127	-0.211	0.464

The sprint results showed that no single study had a dominant impact on the pooled effect size (**Table 7**). The original pooled SMD was -0.07 (95% CI: -0.38 to 0.23). After removing studies one by one, the SMD ranged from -0.18 to 0.01. After removing Castilla-López and Romero-Franco (2023), the pooled effect size became -0.18 (95% CI: -0.50 to 0.15). After removing Kamiş et al. (2024), the effect size direction turned positive but was not statistically significant (SMD = 0.01, 95% CI: -0.33 to 0.34) (**Figure 9d**). In all removal scenarios, the 95% confidence interval included 0, indicating robustness of the negative results, although the removal of some studies had a slight impact on the size and direction of the effect size.

**Table 7:** Leave-one-out sensitivity analysis of sprint performance.

Study omitted	Estimate	95 % Conf. interval	
		Lower CI limit	Upper CI limit
Castilla-López and Romero-Franco (2023)	-0.176	-0.500	0.149
Kamiş et al. (2024)	0.006	-0.326	0.338
Manimmanakorn et al. (2013a)	-0.009	-0.337	0.319
Scott et al. (2017)	-0.083	-0.409	0.243
Scott et al. (2017)	-0.099	-0.426	0.227
Scott et al. (2017)	-0.118	-0.444	0.208
Smith et al. (2025)	-0.054	-0.379	0.272
Smith et al. (2025)	-0.054	-0.379	0.271
Smith et al. (2025)	-0.074	-0.399	0.251
Combined	-0.074	-0.382	0.234

## 4 Discuss

This study revealed a crucial phenomenon for team sports training practices, clarifying the differential effects of BFRT on team-athletes. BFRT significantly induced muscle hypertrophy and increased muscle strength, but failed to additionally improve sprint and jumping performance compared to RT alone. The physiological effects of BFRT may have inherent, task-specific boundaries.

### 4.1 Impact of BFRT on muscle hypertrophy

The results of this meta-analysis indicate that BFRT can significantly induce additional muscle hypertrophy in team-athletes compared to RT alone (p = 0.02). Although the effect size shown in this study is small (SMD = 0.32), the effect has significant biological significance and practical value for athletes with extensive competitive experience (Rhea, 2004). In the context of high-level training, traditional mechanical load stimulation often faces diminishing returns (Lees et al., 2025). BFR induces strong metabolic stress by restricting venous return (Murphy, 2025), including lactate accumulation (Zhou et al., 2024), which in turn triggers a series of combined hormone responses, such as growth hormone (GH) and insulin-like growth factor-1 (IGF-1) (Manini et al., 2012). This metabolic environment simulates the physiological stress of high-intensity training under low mechanical load, effectively promoting protein synthesis by activating the mTOR signaling pathway (Kraemer and Ratamess, 2005). In addition, BFR-induced cell swelling can act as a pressure-sensing signal, activating osmoreceptors on the muscle cell membrane, thus avoiding the high risk of mechanical damage and initiating morphological remodeling (Loenneke et al., 2012).

It is worth noting that the results of this study differ from some previous reviews. For example, Deng et al. (2025) did not observe a significant hypertrophy effect in a meta-analysis of athletes in mixed sports (ES = 0.17, P = 0.293). Another review of similar populations also showed that only 50% of the included studies achieved positive results (Wortman et al., 2021). However, in the literature included in this study on athletes in specific teams, the experimental results of (Scott et al., 2017; Yamanaka et al., 2012; Gjini et al., 2024; Korkmaz et al., 2022; Manimmanakorn et al., 2013a, 2013) are highly consistent with the conclusions of this study.

This difference can be influenced by multiple factors. While some argue that measurement error is the source of the difference, previous studies have presented complex signals. For example, both Korkmaz et al. (2022) and Scott et al. (2017) used ultrasound to measure muscle thickness. The former observed a positive result, while the latter obtained a negative result, suggesting that the measuring tool may not be the only confounding factor. Baseline differences and specific training characteristics among athletes are likely key variables (Reitzner et al., 2024). Previous research has mainly focused on mixed sports with differentiated characteristics, including weightlifting, endurance sports, and team sports. The accumulation of metabolic stress is considered a major factor in BFRT-induced muscle hypertrophy, the intensity of which depends on the intervention dose. Appropriate dosage is key to activating anabolic signaling pathways (e.g., mTORC1) (Loenneke et al., 2017, 2010). However, different studies exhibit high heterogeneity in intervention protocols, which may explain negative results reported in certain athlete populations. REF. Future research should further standardize the gold standard for BFR exercise prescriptions and delve deeper into the specificity of athletes’ adaptation to BFR in different sport contexts.

### 4.2 Impact of BFRT on muscle strength

The results of this meta-analysis indicate that BFRT significantly improves muscle strength in team-athletes (p< 0.001). Similar strength increases were also observed in the study by Yamanaka et al. (2012); Kamiş et al. (2024); Adhitya et al. (2022); Wang et al. (2022); Manimmanakorn et al. (2013a); Manimmanakorn et al. (2013b); Smith et al. (2025); Korkmaz et al. (2022), which was included in this study. This finding is highly consistent with the conclusions of a recent systematic review of athletes by Li et al. (2025), which also confirmed the significant efficacy of BFRT in improving maximum strength (ES = 0.99, P<0.001). They observed a large effect size (ES = 0.99), but its high heterogeneity of 68% suggested significant differences in adaptation across different sports. In contrast, this study focused on team-athletes, and the results showed higher consistency (I^2^ = 10%), providing more robust evidence for strength training in team ball sports.

The underlying mechanism of this phenomenon may lie in the fact that for high-level team-athletes, simple low-load stimulation may not be sufficient to cross their neural drive regulation threshold (Hung et al., 2025). Previous studies have shown that the accumulation of metabolic products and changes in the muscle internal environment stimulate type III/IV afferent nerves, thereby inhibiting the excitability of α motor neurons and recruiting type II motor units with higher thresholds (Blain et al., 2016; Yasuda et al., 2010, 2009). In this context, BFR-induced type II muscle fiber recruitment may be more inclined to improve baseline maximum strength (1RM) rather than instantaneous burst output REF. For team-athletes, using low-load BFRT during the competition season can provide sufficient neural drive stimulation while conserving physical fitness, thereby effectively maintaining or even improving maximum strength.

### 4.3 Impact of BFRT on muscle power

However, the significant increase in muscle strength (SMD = 0.42) did not translate into a synchronous increase in sprint (5-30m) and jumping performance. For high-power output tasks like sprinting and jumping, performance improvement depends on high-power neural drive patterns and specific adaptations to muscle-tendon elasticity (Turner and Jeffreys, 2010; Xu et al., 2025; Lambertz et al., 2003; Toumi and Best, 2005). BFRT emphasizes controlled movement rhythm and metabolic stress, but lacks neural drive stimulation for concentric contraction explosive force and eccentric buffer phase (David M Gundermann et al., 2012; Wang et al., 2025). Our analysis included only one high-load (70% 1RM) study, while the other studies used low loads (20% - 50% 1RM). Under low-load conditions, the low-power output movement patterns of BFRT may provide insufficient stimulus (Luebbers et al., 2014). Athletes’ neuromuscular systems are often already highly adapted, leaving limited room for improvement in explosive power performance (Wernbom et al., 2007). This lack of training specificity may result in muscles failing to optimize their rapid force rate (RFD) during extremely short ground contact times while increasing strength.

It is worth noting that the study by Wang et al. (2022). suggested that when BFR is combined with a sufficiently high load (70% 1RM), it may simultaneously meet the needs of metabolic stress and high neural drive, thereby inducing explosive adaptation. Su et al. (2025) and colleagues obtained similar evidence in their comprehensive study of mixed-sport-athletes. They synthesized evidence from RCTs of BFR combined with high-load training and obtained a significant pooled effect on speed performance (SMD = 0.78, p = 0.03) (Su et al., 2025). Despite the limited supporting evidence, training load may be a key variable regulating the translation of BFR-induced adaptations into explosive power performance (Scott et al., 2016). When BFR is combined with high loads that effectively drive neural adaptation, it may synergistically affect metabolic stress and neuromechanical stimulation, thereby positively influencing power output (Cook et al., 2014). However, high-quality evidence supporting this inference is currently limited. This provides a key hypothesis for future research that the load threshold may be a key regulatory variable in whether BFRT affects neural power adaptation.

In addition, the conversion of strength into explosive power requires a certain period of time (Andersen et al., 2010). The intervention periods in this study were mostly 4–8 weeks, which may be sufficient to induce muscle hypertrophy and improve muscle strength, but not enough to complete the recovery of complex motor skills at higher strength levels, such as sprinting and jumping performance. Therefore, for team-athletes who pursue explosive power, BFRT should be regarded as a means to improve strength reserves, rather than a way to directly improve muscle power.

### 4.4 Practical applications

The observed effect sizes for hypertrophy (SMD = 0.32) and muscle strength (SMD = 0.42) in the current study were small-to-moderate in magnitude. This is consistent with the expectations for highly trained athletic populations, where the ceiling effect often limits the magnitude of physiological adaptations compared to untrained populations (Moberg et al., 2020). In elite sport, even a small effect size (SMD = 0.25) is categorized as the smallest worthwhile change capable of conferring a competitive advantage (Rhea, 2004). Therefore, the modest gains observed here represent a practically meaningful reinforcement of the athletes’ physical foundation.

Those findings demonstrate that BFRT provides additive benefits for muscle hypertrophy and strength even when integrated into the training regimens of well-trained team-athletes. This evidence extends the utility of BFRT beyond its traditional role as a low-load rehabilitative surrogate, positioning it as a potent supplementary stimulus to high-load RT (HL-RT) for breaking through training plateaus. During the Off-season that the Coaches can leverage BFR combined with various loads to maximize the muscle structure and maximal strength. During the competitive season that the low-load BFRT offers a fatigue-stripping strategy, allowing athletes to maintain strength and muscle volume while minimizing joint impact and systemic exhaustion.

However, the observed neutral effects on sprint and jump performance suggest that BFRT should be strategically programmed. For performance transfer that to bridge the gap between strength gains and explosive output, BFRT may be coupled with specialized speed-power or plyometric training to ensure the neural adaptations required for sprinting and jumping.

### 4.5 Limitations

This study has several limitations. First, the exclusion of grey literature may have increased the risk of publication bias to some extent, potentially overestimating the observed effects. Second, although study quality was assessed using the RoB 1.0 tool, the inherent nature of exercise interventions precluded double-blinding in most studies. While this is a common challenge in exercise science, the potential for subjective expectation bias cannot be entirely ruled out. Furthermore, some studies relied on muscle circumference rather than the gold-standard CSA. This may introduce bias in interpreting muscle hypertrophy, as circumference measurements are susceptible to fluctuations in subcutaneous fat and extracellular fluid. Third, most included studies were relatively short-term (4–8 weeks). This duration may capture early neuromuscular adaptations but fails to fully reveal long-term physiological adaptation trajectories, potentially underestimating the cumulative gains of specific BFR protocols. Finally, the observed low heterogeneity (I^2^ = 0 – 10%) should be interpreted with extreme caution. Given each result that fewer than 10 studies were included (k< 10), the statistical power to detect true variation is inherently limited. This statistical homogeneity may mask latent clinical heterogeneity in BFR prescriptions, such as variations in cuff pressure (individualized vs. fixed) and load intensity, which our analysis was not sufficiently powered to capture. Consequently, we refrained from mandatory subgroup analyses to maintain the robustness of the statistical results, though this limits our precise understanding of optimal BFR dosing. Future research should focus on standardizing BFR prescription protocols, employing more precise measurement methods, and extending follow-up periods to explore the long-term physiological responses to BFRT across different athletic levels.

## 5 Conclusion

This evidence suggests that incorporating BFR into RT can yield small-to-moderate positive effects on muscle hypertrophy and strength among team-athletes. While BFRT serves as a viable supplementary strategy for building or maintaining muscular foundations, particularly useful for load management across different seasonal cycles, it does not appear to provide superior benefits for sport-specific explosive performance, such as sprinting and jumping. Given the limited number of included studies and the observed heterogeneity, these findings should be interpreted with caution. Future research with larger sample sizes is required to establish optimal BFR prescriptions for team-athletic.

## Data Availability

The datasets presented in this study can be found in online repositories. The names of the repository/repositories and accession number(s) can be found in the article/**Supplementary Material**.
