# Supplementary material for: Muscle hypertrophy and strength improvements following blood flow restriction combined with resistance training in team-athletes: a systematic review and meta-analysis
Source: Front Physiol. 2026 Jul 20;17:1812707. doi: 10.3389/fphys.2026.1812707 (PMC13430605; doi:10.3389/fphys.2026.1812707)
Supplement: Supplementary file 1 [file DataSheet1.docx]

Supplementary Appendix

**Table of contents**

[Appendix 1: PEDro quality assessment 2](#_Toc23238)

# Appendix 1: PEDro quality assessment

**Table S1. PEDro quality assessment**

**Table.3** PEDro quality assessment of the included studies.

| **References** |  | | | | | | | | | | **Total (from a possible maximal of 10)** |
| --- | --- | --- | --- | --- | --- | --- | --- | --- | --- | --- | --- |
|  | **2** | **3** | **4** | **5** | **6** | **7** | **8** | **9** | **10** | **11** |  |
| Scott et al 2017 | 0 | 1 | 1 | 0 | 0 | 0 | 1 | 1 | 1 | 1 | 6 |
| Castilla-L RoF 2023 | 1 | 1 | 1 | 0 | 0 | 1 | 1 | 1 | 1 | 1 | 8 |
| Yamanaka et al 2012 | 1 | 1 | 1 | 0 | 0 | 0 | 1 | 1 | 1 | 1 | 7 |
| Kamis et al 2024 | 1 | 1 | 1 | 0 | 0 | 0 | 1 | 1 | 1 | 1 | 7 |
| Adhitya et al 2022 | 1 | 1 | 1 | 0 | 0 | 1 | 1 | 1 | 1 | 1 | 8 |
| Gjini et al 2024 | 1 | 1 | 1 | 1 | 1 | 1 | 1 | 1 | 1 | 1 | 10 |
| Wang et al 2022 | 1 | 1 | 1 | 0 | 0 | 0 | 1 | 1 | 1 | 1 | 7 |
| Golubev et al 2021 | 0 | 1 | 1 | 0 | 0 | 0 | 1 | 1 | 1 | 1 | 6 |
| Manimmanakorn et al 2013a | 1 | 1 | 1 | 0 | 0 | 0 | 1 | 1 | 1 | 1 | 7 |
| Manimmanakorn et al 2013b | 1 | 1 | 1 | 0 | 0 | 1 | 1 | 1 | 1 | 1 | 8 |
| Smith et al 2025 | 1 | 0 | 1 | 1 | 0 | 0 | 1 | 1 | 1 | 1 | 7 |
| Korkmaz et al 2022 | 0 | 0 | 1 | 0 | 0 | 1 | 1 | 1 | 1 | 1 | 6 |
| **Median Score =7.25** | | | | | | | | | | | |

1 = criterion is satisfied; 0 = criterion not satisfied
2 = subjects were randomly allocated to groups (in a crossover study, subjects were randomly allocated and order in which treatments were received
3 = allocation was concealed
4 = the groups were similar at baseline regarding the most important prognostic indicators
5 = there was blinding of all subjects
6 = there was blinding of all therapists who administered the therapy
7 = there was blinding of all assessors who measured at least one key outcome
8 = measures of at least one key outcome were obtained from more than 85% of the subjects initially allocated to groups
9 = all subjects from whom outcome measures were available received the treatment or control condition as allocated or, where this was not the case, data for at least one key outcome were analyzed by “intention to treat”
10 = the results of between-group statistical comparisons were reported for at least

one key outcome
11 = the study provided both point measures and measures of variability for at least one key outcome
