# Supplementary material for: Muscle hypertrophy and strength improvements following blood flow restriction combined with resistance training in team-athletes: a systematic review and meta-analysis
Source: Front Physiol. 2026 Jul 20;17:1812707. doi: 10.3389/fphys.2026.1812707 (PMC13430605; doi:10.3389/fphys.2026.1812707)
Supplement: Supplementary file 2 [file DataSheet2.docx]

Supplementary Appendix

**Table of contents**

[Appendix 1: Study characteristics 2](#_Toc23238)

# Appendix 1: Study characteristics

**Table S1. Study characteristics**

| **Study (Year)** | **Sport & Sex** | **Sample Size (EG/CG)** | **Age (EG / CG)** | **Exercise Protocol (Type, Duration, Freq, Load)** | **BFR Parameters (Pressure, Width, Mode)** | **Control** | **Outcomes** |
| --- | --- | --- | --- | --- | --- | --- | --- |
| Scott et al 2017 | Football, M | 18(10/8) | 19.8 ± 1.5/  19.8 ± 1.5 | Squat (5 weeks, 3x/w),  4 sets (15–30) @20–30% 1RM | 7/10 PP, 7.5cm, Con-BFR | LL | CMJ↔, 10m↑, 20m↔, 40m↔,Muscle thickness↑ |
| Castilla-L and Romero-F 2023 | Soccer, M | 18(NR) | 19.22 ± 1.69/  19.22 ± 1.69 | Squat (6 weeks, 2x/w),  4 sets (15) @ 20-35% 1RM | 40-80% AOP, 7cm, Int-BFR | HL | Girth↑, MVC↔, CMJ ↔, 30m↑ |
| Yamanaka et al 2012 | Football, NR | 32(NR) | 19.2 ± 1.8/  19.2 ± 1.8 | Bench press & Squat (4 weeks, 3x/w),  4sets (20-30) **@** 20%1RM | NR, 5cm, Con-BFR | No-BFR | 1RM(Squat)↑, Girth(R/L)↑ |
| Kamis et al 2024 | Football, NR | 24(12/12) | 19.25 ± 0.86/  19.42 ± 1.24 | Squat (8 weeks, 3x/w),  4sets (30-15-15-15) @20%1RM | 40%-80%LOP, 10.5cm, Con‐BFR | No-BFR | Peak power↑, CMJ↔, 30m↑ |
| Adhitya et al 2022 | Basketball/rugby, NR | 43(23/20) | 16.3 ± 1.4/  17.1 ± 2.3 | Squat (8 weeks, 2x/w),  4 sets（10）@ 30%1RM | 70%AOP, NR, Int‐BFR | No-BFR | Quadriceps strength↑ |
| Gjini et al 2024 | Basketball, NR | 18(9/9) | 21.44 ± 4.90/  19.00 ± 3.96 | Plantar flexion (4 weeks, 3x/w),  3 sets (3) @40%1RM | 200mmHg, NR, Int‐BFR | No-BFR | Perimeter(R/L)↔, VJ↑ |
| Wang et al 2022 | Volleyball, M | 12(6/6) | 20.50 ± 1.38/  20.83 ± 1.47 | Squat (8 weeks, 3x/w),  4 sets (30-15-15-15) @30%1RM | 50%AOP, 7cm, Con‐BFR | HL-RT | SJ↔ |
|  |  | 12(6/6) | 20.17 ± 0.75/  20.83 ± 1.47 | Squat (8 weeks, 3x/w),  4 sets (30-15-15-15) @ 70%1RM |  |  | 1RM(Half-squat) ↑, SJ↑ |
| Golubev et al 2021 | Volleyball, M | 18(9/9) | 18.7±0.5/  18.7 ± 0.5 | Knee Extension & Squat (NR, 2x/w),  3 sets @25-40%1RM | 400 SCU, 5cm, Con‐BFR | No-BFR | MVC↔ |
| Manimmanakorn et al 2013a | Football, F | 20(10/10) | 20.2 ± 3.3/  20.2 ± 3.3 | Knee extension & flexion (5 weeks, 3x/w),  6 sets @20%1RM | 230mmHg, 5cm, Con‐BFR | No-BFR | CSA↑, MVC3↑, VJ↑, 10m↔ |
| Manimmanakorn et al 2013b | Basketball, F | 20(10/10) | 20.2 ± 3.3/  20.2 ± 3.3 | Knee extension & flexion (5 weeks, 3x/w),  6 sets @20%1RM | 230mmHg, 5cm, Con‐BFR | No-BFR | CSA↑, MVC3↑ |
| Smith et al 2025 | Basketball, F/M | 17(9/8) | 21.1 ± 1.5/  22.0 ± 2.1 | Hard draw & Squat (4 weeks, 3x/w),  3 sets (10) @25-30%1RM | 20-60%AOP, 10cm, Int‐BFR | No-BFR | Squat↑, CMJ↑, 5m↑, 10m↑, 20m↔ |
| Korkmaz et al 2022 | Football/F | 23(11/12) | 18.36 ± 0.5/  18.42 ± 0.79 | Quadriceps extension (6 weeks, 2x/w),  4 sets (30-15-15-15) @30%1RM | 130–150mmHg, 7cm, Con‐BFR | No‐BFRHL | Muscle thickness↑, Peak knee torque↑ |
| Note: EG = Experimental Group; CG = Control Group; M = Male; F = Female; NR = Not Reported; BFR = Blood Flow Restriction; Con-BFR = Continuous BFR; Int-BFR = Intermittent BFR; AOP = Arterial Occlusion Pressure; LOP = Leg Occlusion Pressure; PP = Perceived Pressure; LL = Low Load; HL = High Load; CMJ = Countermovement Jump; VJ = Vertical jump; SJ= Squat Jump; MVC = Maximal Voluntary Contraction;1RM = One-repetition maximum; CSA = Cross-sectional area;↑ indicates significant improvement; ↔ indicates no significant difference compared to baseline or control. | | | | | | | |
