# Supplementary material for: Muscle hypertrophy and strength improvements following blood flow restriction combined with resistance training in team-athletes: a systematic review and meta-analysis
Source: Front Physiol. 2026 Jul 20;17:1812707. doi: 10.3389/fphys.2026.1812707 (PMC13430605; doi:10.3389/fphys.2026.1812707)
Supplement: Supplementary file 3 [file DataSheet3.docx]

Supplementary Appendix

**Table of contents**

[Appendix 1: Muscle hypertrophy 2](#_Toc23238)

[Appendix 2: Muscle strength 3](#_Toc23238)

[Appendix 3: Jump performance 4](#_Toc23238)

[Appendix 4: Sprint performance 5](#_Toc23238)

# Appendix 1: Muscle hypertrophy

**Fig S1. Funnel chart, Egger chart、Leave-one-out sensitivity analysis of Muscle hypertrophy**

|  |
| --- |
| **Egger's test: P=0.35**   |
| 15. |

Table 4. Leave-one-out sensitivity analysis for muscle hypertrophy

| **Study omitted** | **Estimate** | **95% Conf. Interval** | |
| --- | --- | --- | --- |
|  |  | **Lower CI Limit** | **Upper CI Limit** |
| Castilla RF et al (2023) | 0.349 | 0.081 | 0.617 |
| Gjini et al A (2024) | 0.363 | 0.095 | 0.631 |
| Gjini et al B (2024) | 0.351 | 0.083 | 0.619 |
| Korkmaz et al A (2022) | 0.365 | 0.094 | 0.636 |
| Korkmaz et al B (2022) | 0.284 | 0.014 | 0.554 |
| Manimmanakorn A a (2013) | 0.269 | 0.002 | 0.537 |
| Manimmanakorn B a (2013) | 0.274 | 0.006 | 0.541 |
| Manimmanakorn A b (2013) | 0.274 | 0.006 | 0.542 |
| Scott et al (2017) | 0.378 | 0.110 | 0.646 |
| Yamanaka et al A (2012) | 0.358 | 0.081 | 0.635 |
| Yamanaka et al B (2012) | 0.359 | 0.082 | 0.636 |
| Combined | 0.329 | 0.072 | 0.586 |

# Appendix 2: Muscle strength

**Fig S2. Funnel chart, Egger chart、Leave-one-out sensitivity analysis of Muscle strength**

| 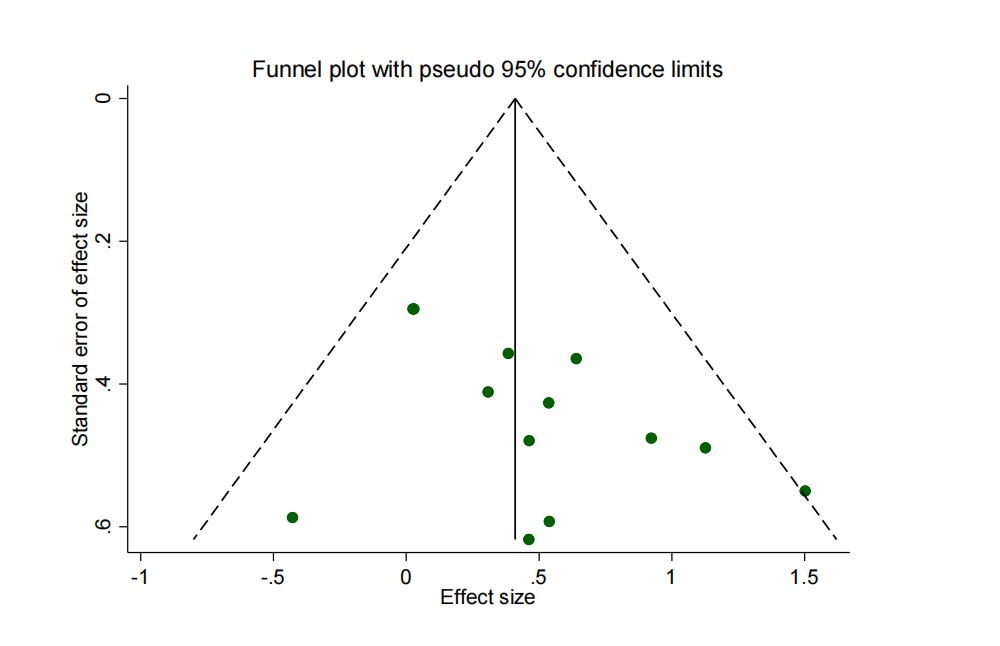 |
| --- |
| 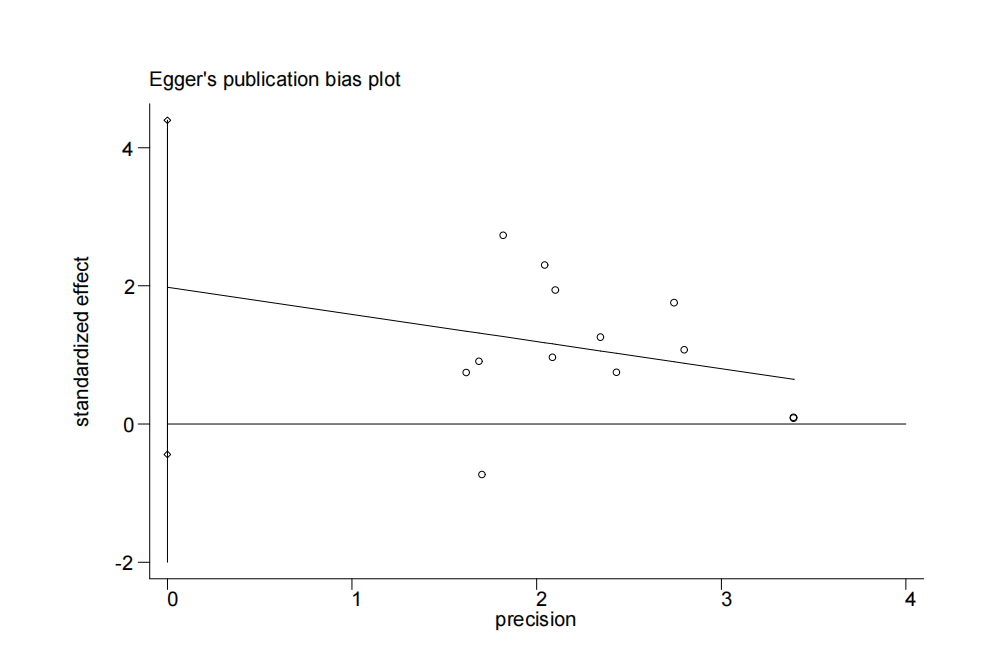  **Egger's test: P=0.099** |
|  |

Table 5. Leave-one-out sensitivity analysis of muscle strength

| **Study omitted** | **Estimate** | **95% Conf. Interval** | |
| --- | --- | --- | --- |
|  |  | **Lower CI Limit** | **Upper CI Limit** |
| Adhitya et al A (2022) | 0.514 | 0.247 | 0.781 |
| Adhitya et al B (2022) | 0.514 | 0.247 | 0.781 |
| Castilla RF et al (2023) | 0.458 | 0.181 | 0.736 |
| Golubev et al (2021) | 0.374 | 0.141 | 0.606 |
| Kamis et al (2024) | 0.473 | 0.192 | 0.754 |
| Korkmaz et al A (2022) | 0.452 | 0.172 | 0.732 |
| Korkmaz et al B (2022) | 0.437 | 0.157 | 0.717 |
| Manimmanakorn a (2013) | 0.416 | 0.153 | 0.679 |
| Manimmanakorn b (2013) | 0.395 | 0.146 | 0.644 |
| Smith et al (2025) | 0.458 | 0.184 | 0.731 |
| Wang et al A (2022) | 0.482 | 0.234 | 0.730 |
| Wang et al B (2022) | 0.453 | 0.180 | 0.727 |
| Yamanaka et al (2012) | 0.469 | 0.184 | 0.754 |
| Combined | 0.453 | 0.196 | 0.709 |

Appendix 3: **Jump performance**

**Fig S3. Funnel chart, Egger chart、Leave-one-out sensitivity analysis of Jump performance**

|  |
| --- |
|   **Egger's test: P=0.932** |
|  |

Table 6. Leave-one-out sensitivity analysis of jump performance

| **Study omitted** | **Estimate** | **95% Conf. Interval** | |
| --- | --- | --- | --- |
|  |  | **Lower CI Limit** | **Upper CI Limit** |
| Castilla RF et al (2023) | 0.142 | -0.220 | 0.504 |
| Gjini et al (2024) | 0.194 | -0.168 | 0.556 |
| Kamis et al (2024) | 0.061 | -0.310 | 0.432 |
| Manimmanakorn a (2013) | 0.054 | -0.311 | 0.418 |
| Scott et al (2017) | 0.205 | -0.157 | 0.566 |
| Smith et al (2025) | 0.138 | -0.223 | 0.499 |
| Wang et al A (2022) | 0.162 | -0.191 | 0.515 |
| Wang et al B (2022) | 0.055 | -0.296 | 0.407 |
| Combined | 0.127 | -0.211 | 0.464 |

# Appendix 4: Sprint performance

**Fig S4. Funnel chart, Egger chart、Leave-one-out sensitivity analysis of Sprint performance**

|  |
| --- |
|   **Egger's test: P=0.060** |
|  |

Table 7. Leave-one-out sensitivity analysis of sprint performance

| **Study omitted** | **Estimate** | **95% Conf. Interval** | |
| --- | --- | --- | --- |
|  |  | **Lower CI Limit** | **Upper CI Limit** |
| Castilla RF et al (2023) | -0.176 | -0.500 | 0.149 |
| Kamis et al (2024) | 0.006 | -0.326 | 0.338 |
| Manimmanakorn a (2013) | -0.009 | -0.337 | 0.319 |
| Scott et al A (2017) | -0.083 | -0.409 | 0.243 |
| Scott et al B (2017) | -0.099 | -0.426 | 0.227 |
| Scott et al C (2017) | -0.118 | -0.444 | 0.208 |
| Smith et al A (2025) | -0.054 | -0.379 | 0.272 |
| Smith et al B (2025) | -0.054 | -0.379 | 0.271 |
| Smith et al C (2025) | -0.074 | -0.399 | 0.251 |
| Combined | -0.074 | -0.382 | 0.234 |
